# Mathematical Methods for Assessing the Prognostic of Fixed Partial Dentures Resulting from Evaluating a Group of Dental Patients in Romania

**DOI:** 10.1155/2014/984901

**Published:** 2014-06-09

**Authors:** Ioana Chifor, Alexandru I. Mitrea, Iulia Clara Badea, Radu Chifor, Mindra Eugenia Badea, Paulina Mitrea, Sever Popa, Maria Crisan, Ramona Avram

**Affiliations:** ^1^Department of Preventive Dentistry, Iuliu Hatieganu University of Medicine and Pharmacy of Cluj-Napoca, Victor Babeş Street, No. 15, 400012 Cluj-Napoca, Romania; ^2^Faculty of Automation and Computer Science, Technical University of Cluj-Napoca, George Baritiu Street, No. 26-28, 400027 Cluj-Napoca, Romania

## Abstract

Based on some mathematical and statistical approaches, our study leads to some conclusions concerning the procedures related to the orodental prosthetics. Occlusal equilibration in orodental prosthetics is a major issue because besides motivating patients for a regular daily oral hygiene, it could significantly increase the longevity of FPR. More dental hygiene information should be given after prosthetic treatment and patients should be motivated to attend recalls on a regular basis for professional teeth-cleaning. Interdental cleaning aids should be explained and the patients have to be motivated to use them at least once a day and the using technique should be individualized. Regarding the application of the deformable models theory, implemented in the context of an expert type software environment, it is known that the fact that modelling by advanced methods and techniques based on the deformable surfaces theory increases the efficiency of the dentofacial prosthetics procedures is a domain of great interest in the actual medical research.

## 1. Introduction

The prognosis and the success of fixed dental prosthesis (FDP) are calculated most often by self-assessment of the oral health related quality of life of the patients.

Some of the noninvasive treatments, such as Resin bonded bridgeworks (RBB), are often overlooked by practitioners despite a large amount of evidence supporting the technique. For example, in Cork University Dental School, an evidence-based study evaluated the success of the standardized approach on the delivery of RBB by students. The authors reviewed 222 bridges which had been delivered over a 6-year time period between 2002 and 2007. A success rate of 84.1% was achieved, with a mean survival time of 41 months. This study illustrates that predictable and highly successful RBB can be delivered even by inexperienced clinicians using an evidence-based, standardized approach [1].

Quality of life related to oral health is difficult to be assessed and moreover it is still sometimes incoherent with the actual clinical status. “The oral problem count per day that corresponds to one Oral Health Impact Profile-49 point can be used to interpret this instrument's scores in cross-sectional and longitudinal studies. This number can help to better understand OHRQoL burden for patients, clinicians, and researchers alike” [2].

Therefore, another approach should be sought for choosing the best treatment option and for assessing the benefits and the flaws of a type of treatment.

The survival of fixed dental prosthesis (FDP) is defined usually by the bridgework being in use after a number of years: from 5 years according to [3] to 12 years according to [4] as acceptable limits before replacing it.

Despite the constant decreasing of caries prevalence in childhood, the risk for adults to develop different orodental diseases is constant or slightly increasing.

The Global Burden of Disease (GBD) 2010 Study produced comparable estimates of the burden of 291 diseases and injuries in 1990, 2005, and 2010. This paper reports on the global burden of untreated caries, severe periodontitis, and severe tooth loss in 2010 and compares those figures with new estimates for 1990. Marcenes et al. [5] used disability-adjusted life-years (DALYs) and years lived with disability (YLDs) metrics to quantify burden. Oral conditions affected 3.9 billion people, and untreated caries in permanent teeth were the most prevalent condition evaluated for the entire GBD 2010 Study (global prevalence of 35% for all ages combined). Oral conditions combined accounted for 15 million DALYs globally (1.9% of all YLDs; 0.6% of all DALYs), implying an average health loss of 224 years per 100,000 population. DALYs due to oral conditions increased 20.8% between 1990 and 2010, mainly due to population growth and aging. While DALYs due to severe periodontitis and untreated caries increased, those due to severe tooth loss decreased. DALYs differed by age groups and regions but not by genders. The findings highlight the challenge in responding to the diversity of urgent oral health needs worldwide, particularly in developing communities [5].

Even in countries where intensive preventive care is performed, the overall oral health of adults is not improving. This is proved by several studies, amongst which the Danish Health Examination Survey (DANHES 2007-2008) aimed “(1) to establish an oral health for adult Danes and (2) to explore the influence of general diseases and lifestyle on oral health.” The study population comprised 4402 subjects, aged 18–96, consecutively enrolled from 18 065 DANHES participants from 13 municipalities in Denmark. The oral part consisted of a validated questionnaire and a clinical examination, carried out in mobile units by three trained and calibrated dental hygienists. The data were processed with descriptive statistics and mono- and bivariate analyses. The mean age was 54.1 years and 60% were women. The mean number of natural teeth was 26.6; the mean decayed, missing, filled teeth (DMFT)/decayed, missing, filled surfaces (DMFS) values were 18.9 and 61.0 and varied with age (DMFT 8.7–24.3). A higher proportion of females suffered from dental erosion in the younger age groups. Forty percent of all subjects had a mean clinical attachment loss ≥ 3 mm, varying from 4% among those aged 18–34 to 80% in those over 75. A suboptimal saliva secretion rate was more common among females than males (17.7% versus 10.4%) and this was reflected by the reported frequency of dry mouth. This extensive cross-sectional study provides a platform for obtaining future knowledge of the impact of health- and lifestyle-related factors on oral diseases [7].

Assessing the longevity of dental restoration is very difficult due to the various confusing factors that arise, such as individual oral hygiene, appropriate design and quality of the initial restorations, quality of occlusion, parafunctions, dental recalls attended, associated general diseases that may influence the periodontal support, and/or the salivary flow and caries risk, [8, 9].

In 2007, Güngör et al. [10] focused on overall clinical performance during 7 years, determined by using modified United States Public Health Services criteria and evaluated with Kaplan-Meier survival analysis.

The extensive search we performed on Medline PubMed and in the available literature for* articles with similar evaluation criteria for the causes of failure of FPR identified nine possible causes of failure described in* studies [8, 11] with similar evaluation criteria, of which we took the results published by Goodacre et al. [8] in 2003, is a reference point for the failure causes (Table 1).

**Figure 1:**
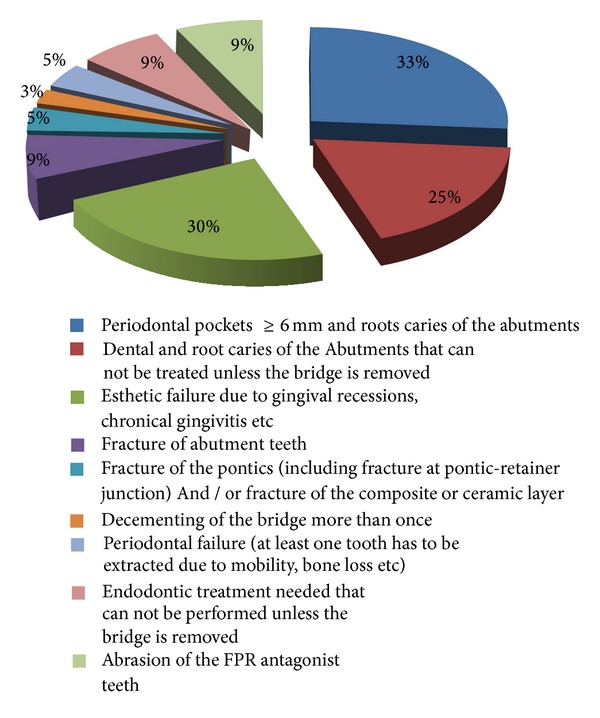
Main failure cause of dental bridges at the time of their removal [6].

In a previous retrospective survey we performed in 2009-2010, we identified the failure cause for FPR at the time of their removal. All the examiners were calibrated to assess the previously identified nine failure causes (Table 1) and to calculate the Plaque Index (after O'Leary). At the end of calibration, the interexaminer kappa was >80% for all items. The study included the 45 patients who asked for dental treatment because of a tooth-supported FPR failure and who had this FPR made in the previous eight years. An informed consent of each patient was obtained. The study included all the patients from the dental offices selected for the study, for which the dentists decided that there was indeed a need of replacement of the tooth-supported FPR. All the patients agreed to take part in the survey. The questionnaire was validated through a pilot study with 2 dentists and 20 patients. The Plaque Index of the patients concerned was recorded by the dentists, on the survey form, according to the O'Leary method (percentage of surfaces with plaque deposits), after the patient was asked to chew a plaque-disclosing tablet for 3 minutes and then to rinse. The survey form had six items filled in by the patient, regarding sociodemographic data (open questions) and the following closed questions (multiple choice questions with more than one answer possible): symptoms regarding the FPR abutments and surrounding gingival tissue, oral hygiene information received after the initial treatment, oral hygiene knowledge regarding FPR cleaning, daily individual hygiene habits, and recall visits attended (frequency and reasons). The dentists filled in an item regarding the type of FPR failure (of the nine causes; more than one answer are possible) and the treatment they choose to perform. A complete dental chart was recorded, on which the dentists noted for the FPR abutments the dental caries according to the International Caries Detection and Assessment System II 2005 at cavity level (scores 3 to 6). The dentists recorded the periodontal pockets depth in 6 points (mesiomouth cavity, mouth cavity, distomouth cavity, distolingual, lingual, and mesiolingual). Statistical analysis was performed using the SPSS (version 13.0) statistical package and the Microsoft Office Excel 2007. Descriptive and multivariate regression analysis was employed. The logistic regression model was used to assess the relationship between failure causes, number of missing teeth, the presence of interference and premature contacts, oral hygiene behavior, and knowledge about oral hygiene and attitude towards this and towards the dental visits [6]. We found that RPF needed to be replaced due to 6 mm pockets associated with at least I/II degree mobility at abutments in 12 subjects (26.67%), dental caries at 8 patients (17.77%), and aesthetic failure for 11 patients (24.44%)—Figure 1.

The main failure causes shown in Figure 1 were mostly often associated with defficiencies in design and execution of the FPR for 7 patients (15.55%) and with parafunctional occlusal issues associated with FPR in 8 patients (17.77%).

The mean ICDAS values recorded were 6.57/5.13 (SD = ±3.82/2.16) with an average D3MF-S index of 36.7 (*D* = 11.48, *M* = 12.52, *F* = 12.70).

33 subjects (73.33%) said they have not received any individual dental hygiene information regarding interdental cleaning aids (IDCAs) for the maintenance of their FPR after their initial treatment.

**Figure 2:**
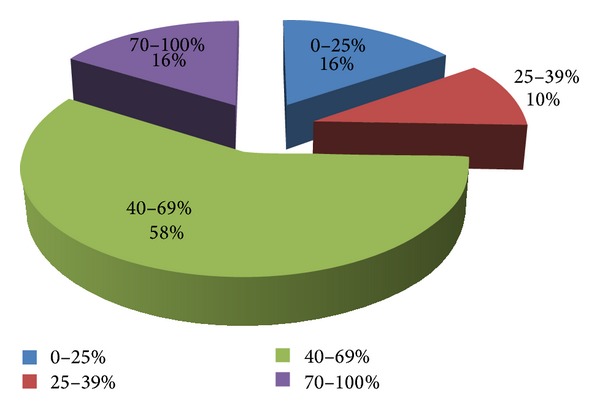
Plaque index at a group with FPR failure [6].

10 patients (22.22%) reported that they brushed thier teeth once a day. 9 patients (20%) declared that even if they were informed regarding the necessity of daily use of auxiliary oral hygiene methods and IDCAs, they performed just the toothbrushing 2-3 times a day. Only 11 patients (24.44%) used daily at least one of the IDCAs, which explains the distribution of the plaque index—Figure 2.

Less than 10% (just 4 patients) had a checkup once a year, whereas the rest of them asked for an appointment only because of pain or aesthetic problems from their FPR. The multivariate multilevel logistic regression model used to assess the relationship between failure causes, average income, knowledge and daily habits of oral hygiene, and attitude towards the dental visits showed a statistically significant influence of the above mentioned explanatory factors on the failure causes related to dental and root caries (predicting the localization of caries on proximal surfaces and their depth) and on the number of missing teeth, the depth of periodontal pockets at the abutment teeth (*P* < 0.05) [6].

The variable “functional occlusion” was a factorization characteristic, resulting in 2 subgroups according to values YES/NO. In the two subgroups, the study of univariate association between “Lifespan of FPR” and “frecquency of use of IDCAs” and “Lifespan of FPR” and “material of FPR” according to the cumulative role of “frecquency of using IDCAs” and “material of FPR”, by bivariate regression, did not show a statistically significant influence in the studied group (*P* > 0.05) [6].

## 2. Aims and Objectives

We consider that the treatment protocols should be better evaluated and the approach should be shifted so that more dental treatment options could be considered for each clinical case. That is why our aim was to find an evidence-based assessment method for every fixed dental prosthesis (FDP) that could be indicated in each clinical case.

The main objective of our study was to build up an algorithm for predicting the probability of failure of fixed prosthetic restorations (FPR) based on assessing the relationships between oral hygiene (information received about oral hygiene procedures, daily oral hygiene behavior), the number of missing teeth, the functional occlusion, the socioeconomic status, and the causes of failure of FPR from previous studies. This algorithm was implemented in the context of a dedicated software environment, named MoDef, which we developed in order to be able to combine both the deformable model mathematical theory based assessment and the statistical followup (see Figure 8).

## 3. Material and Methods

**Figure 3:**
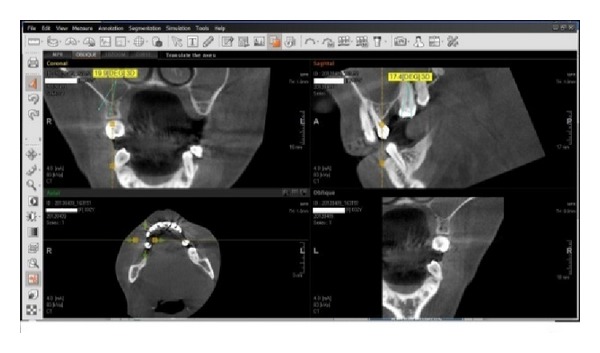
CBCT.

In order to define the input and output parameters of the software for simulating the dental bridges biodynamic which we implemented based on the deformable models mathematical theory, we have studied classical and digital retroalveolar and bite-wing dental X-rays, ortopantomografias (OPT), and 3D examinations, Cone Beam Computer Tomographies (CBCT)—Figure 3.

In order to verify on the clinical cases the frequency of failure causes previously identified, we used the device meant to measure the dental mobility, Periotest *C* (Medizintechnik Gulden) of the Dental Prevention Department from Cluj-Napoca. It returns scores between −8 and +50 according to the mobility of the tooth tested by applying the percussion head on the buccal surface. The higher the stability/resistance of the tooth which is tested, the lower the score showed by the Periotest *C* (Table 2).

Based on these previous data, we searched for a function that could model the failure probability and we defined the parameters for an algorithm for FPR prognosis.

## 4. Results 

Based on the data obtained from the literature review and from the clinical studies, we suggest the following steps for simulating FPR biodynamics.

**Figure 4:**
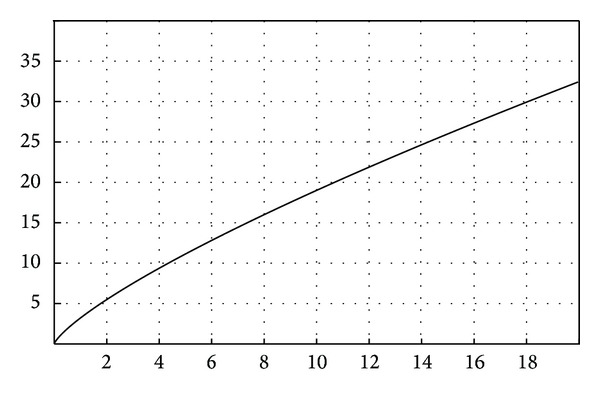
FPR failure probability function.

**Figure 5:**
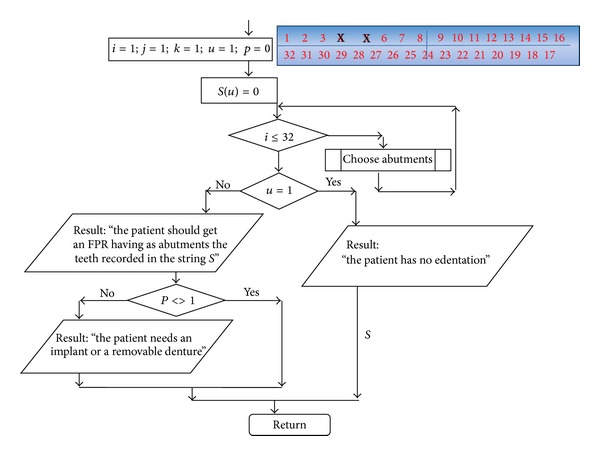
Main structure of the algorithm for FPR type selection.

**Figure 6:**
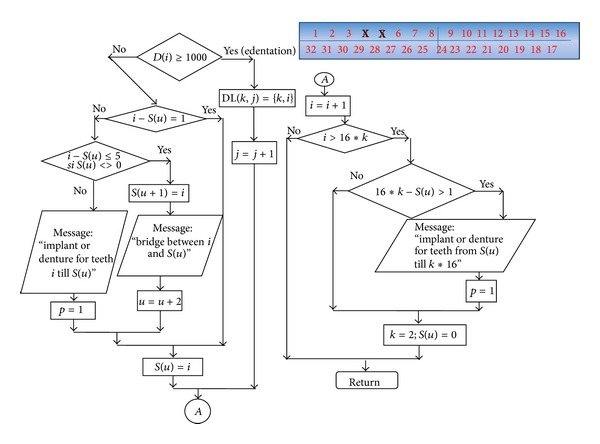
The structure for teeth selection procedure.

1. The function for failure probability (% of failure according to the time) resulted from the meta-analysis—fESEC (Figure 4);
2. the algorithm for selecting the abutment teeth of FPD as a part of determining the failure probability (KTOT) for each individual clinical case (Figures 5 and 6);
3. method for calculating KTOT (Tables 3, 4, 5, 6, 7, 8, and 9);
4. obtaining the set of 3 points for generating the function for FDP failure probability of the studied patient (fPATIENT);
5. graphical representation of the function fPATIENT at any time (Figure 10);
6. identifying the probability of occurrence for the main failure causes c1⋯c9 as a method to represent FDP biodynamics (Table 11).

The general “function” for the “failure” of FPD FPR is *f*(*x*) = 3.210817905∗*x* ^∧^(0.7723530507).

The failure probability function was generated based on 3 sets of values: (0, 0), (10, 18)—knowing from the meta-analysis that the failure probability after 8 years is 16% according to Quinn et al. [12]—the minimal survival rate after 10 years of a number of 248 bridges, and (15, 26)—knowing from the meta-analysis that the failure probability after 15 years is 26% according to Creugers on 4118 bridges [6].

### 4.1. Calculation Method for KTOT

Therefore, we used the following formula for calculating each component of KTOT:

(1) S=W1+W2+⋯+W10,

where *W*1: *S*, *K*1: 100, and *K*1 = 100∗*W*1/*S*.

According to the coefficients calculated based on the 2 previous Tables 8 and 9, we calculated

(2) KTOT=(a1∗7.15+a2∗8.33+a3  +a4∗6.75+a5∗9.92+a6∗12.3  +a7∗12.3+a8∗8.73+a9∗11.11  +a10∗12.3)×(100)−1,KTOT=(−25∗7.15+20∗8.33+39∗11.11  +25∗6.75+33∗9.92+33∗12.3  +0∗12.3+0∗8.73+0∗11.11  +66∗12.3)×(100)−1=21.35%.

For the above mentioned example, the individualised function for fail FPR is represented in Figure 9:

 fesec(0.5) = 1.88

The failure probability after 6 months: 1.88%.

The function was generated by mathematical regression based on 3 sets of values:

 (0, 0)
 (0.5, 1.88)
 (8, 21.35)

Probability of failure of a FDP due to each of the causes c1–c9 may be estimated (Table 11) according to their prevalence at 8 years, as we identified from the clinical study (Figure 1).

## 5. Discussions

We consider that the FPR longevity is very difficult to assess since there are lots of confusing factors which are interrelated.

From the extensive search we did in the medical literature, to our knowledge, this is the first publication regarding the relationship between oral hygiene behavior and the causes of failure of FPR. On an intuitive level, poor oral hygiene and the lack of interdental cleaning aids were expected to be associated with failure due to caries, periodontitis, deficiencies of design and execution of FPR, and the associated parafunctions or their complications, but the association needs further investigations, especially for clinical cases with a functional occlusion.

In order to verify this association, we consider that necessary study should further investigate this possible influence on a larger number of patients and on a wider geographical area.

A possible source of bias in the present study is that it is a retrospective evaluation, based also on anamnesis' data, but we consider that due to the large time span between the initial treatment and the moment of failure of the FPR; planning a prospective study would be unrealistic since the number of patients lost to followup would be extremely high especially due to the particular conditions of dental treatment in Romania (the fact that the patients need to pay themselves the costs of dental treatments) which probably counts for the reduced frequency of asking for regular dental care.

We consider that it would be interesting to assess the influence of root and interproximal caries on the endodontic problems and need for endodontic treatment (including cases when the endodontic treatment can be performed without the removal of FPR).

During the clinical exam, we noticed deficiencies of design and execution of FPR that created retentive areas that made the cleaning more difficult.

Among possible confusing factors that we consider to be very difficult to assess and which are a major source of error are the following two. First at the time of the FPR removal, it is almost impossible to evaluate if there were initially any dental caries in the abutments and especially if they were correctly treated before the prosthetic treatment. Another problem is the effect of prosthetic preparation on the vitality of pulp tissues (in the present study we tried to identify anamnesis, using standard questions, relevant symptoms for hiperemia and for partial pulpitis immediately after the application of FPR), but we consider that due to the very long time between the initial treatment and the moment of failure, there is a large source of error in collecting these information (see Table 10).

**Figure 7:**
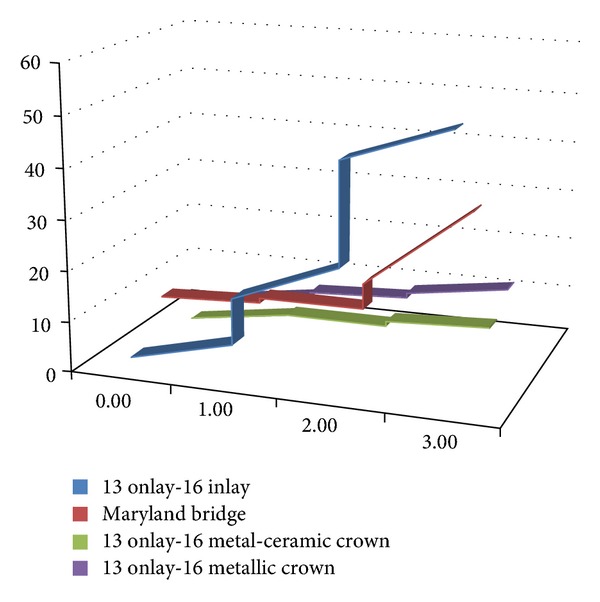
Example of graphic regarding the probability of failure (%) for different types of RPF.

**Figure 8:**
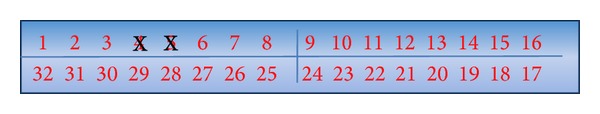
Example of clinical case where 14 and 15 (according to FDI) were missing.

**Figure 9:**
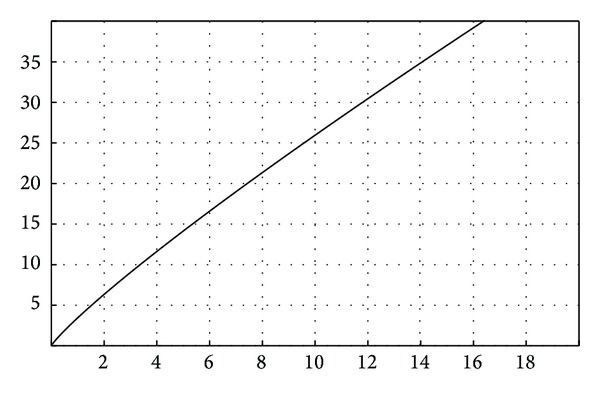
Example of the probability of failure function for the case AB.

**Figure 10:**
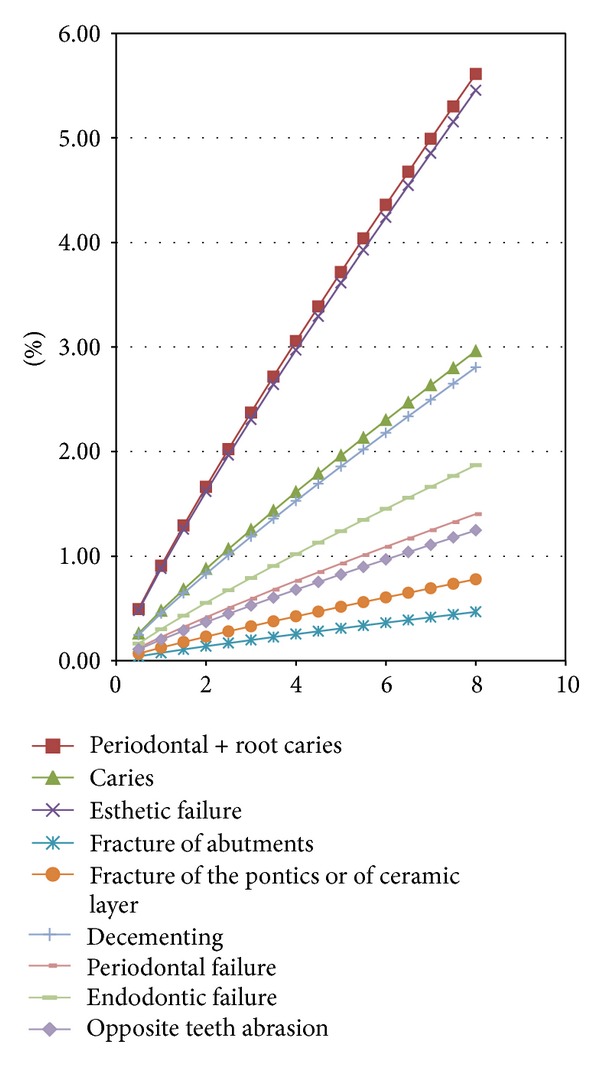
Prognostic function ilustrates the probability of failure over time according to each cause.

Tighter correlations between oral hygiene and associated failure causes (deficiencies of design and execution associated with parafunctions and periodontal pockets of 4–6 mm associated with root caries at abutments) compared to those between oral hygiene and singular failure causes suggest that a bad oral hygiene may determine the necessity of sooner replacement of FPR, but a carefully planned study should do further investigations. We consider that such a study could be planned starting from the same criteria used for assessing the odontal restorations longevity. It would be interesting to assess the relation between FPR longevity, failure causes, and favorable or determinant risk factors used in this study. The survival analysis with Cox or Kaplan-Meier models may bring along proofs for such a relationship, if this study could be performed on a larger group of patients (see Figure 7).

## 6. The Assessment of the Quality of Dental Prosthesis by Methods Based on Deformable Models 

### 6.1. The Notion of Active Deformable Surface

Given the unit square *D* = [0,1]×[0,1], let *S* be a surface defined by the vectorial function *v* : *D* → *R* ^3^, *v* = (*v*_1_, *v*_2_, *v*_3_), (*S*) : *v* = *v*(*s*, *r*); that is, *x* = *v*_1_(*s*, *r*), *y* = *v*_2_(*s*, *r*), *z* = *v*_3_(*s*, *r*). Denote by *A* the class of admissible surfaces *v* ∈ *C* ^4^(*D*, *R* ^3^), whose values on the border of *D* are given.

Suppose that the following data are given, too: the real function *I* = *I*(*v*(*s*, *r*)) = *I*(*x*, *y*, *z*) of class *C* ^2^(*R* ^3^), named* image intensity*, the real function *P*(*v*(*s*, *r*)) = − ||∇*I*(*v*(*s*,*r*)||^2^ which gives the* potential* associated with the external forces, the* elasticity coefficients* (*w*_10_ si *w*_01_), the* rigidity coefficients* (*w*_20_ si *w*_02_), and the* coefficient of resistance to twist* (*w*_11_), associated with the surface (*S*). Now, introduce the* energy-functional E* : *A* → *R* by

(3) E(v)=∫D(w10||vs||2+w01||vr||2  +2w11||vsr||2+w20||vss||2  +w02||vrr||2+P(v(s,r)))ds dr,

where *v*_*s*_, *v*_*r*_ are the partial derivatives of first order of the vectorial function *v* and *v*_*ss*_, *v*_*sr*_, *v*_*rr*_ are the second partial derivatives of *v*. The triple (*S*, *I*, *E*) is said to be an* active deformable surface.*

The minimum of the energy-functional is determined according to Euler-Lagrange-Gauss-Ostrogradsky equations of calculus of variations:

(4) (ELGO)w10vss+w01vrr+F(v)  =w20vssss+w02vrrrr+2w11vsrsr

which describe, from mathematical point of view, a system of partial differential equations; the meaning of *F* is *F*(*v*) = −∇*P*(*v*).

We associate to (ELGO) the so-called* evolution equation* of the surface *S*, in which we add a temporal parameter to the vectorial function *v*; that is, *v* = *v*(*t*, *r*, *s*):

(5) vt−w10vss−w01vrr+w20vssss   +w02vrrrr+2w11vsrsr=F(v),

together with an initial estimation of *S*, namely, *v*(0, *r*, *s*) = *v*_0_(*r*, *s*), and corresponding boundary conditions.

A solution of this static problem is found when the solution *v*(*t*, *r*, *s*) uniformly converges as *t* tends to infinity [13, 14].

In order to solve the system of partial differential equations (ELGO), we make use of discrete iterative methods such as finite differences method or finite-element method. 

*The Finite Differences Method.* This method leads to a linear system of the form:

(6) (Vt−Vt−1)τ+AVt=F(Vt),

where *τ* is the time step, *V* ^*t*^ is the vector whose components are the values of *v* at the nodes of discretization at iteration *t* (*V* ^0^ is given by the initial estimation), and *A* is a pentadiagonal given matrix. Since the unknown *V* ^*t*^ appears in the three terms of the previous equation, we say that this scheme is totally implicit, which leads to complicate calculus, since the force *F* has a complicated form. In this situation, we approximate *V* ^*t*^ by *V* ^*t*−1^ in the terms of *F*(*V* ^*t*^), so we obtain a semi-implicit scheme which gives the following expression for *V* ^*t*^ : *V* ^*t*^ = (*I* − *τA*)  *V* ^*t*−1^ + *τF*(*V* ^*t*−1^), where *I* is the identity matrix.

*The Finite-Element Method*. Firstly, we define the associated problem of the system of partial differential equations (ELGO), secondly we pass to its discrete variant by means of Ritz-Galerkin type methods, and finally we construct the subspace *V*_*h*_ associated to the corresponding Sobolev space making use of elements of Bogner-Fox-Schmit type.

### 6.2. Dental Prosthetics Assessment Computerized Modelling 

Based on this mathematical foundation presented in Section 6.1 we provide computerized assistance in performing trusted dental prosthetics solutions and their quality dual assessment, made by combining both deformable model based algorithms and the statistical assessment presented in the previous sections. The software implementation is made in the context of the MoDef software environment, where it is also performed, starting from the morphological characteristics of each patient, a computerized three-dimensional virtual model, which reproduce the anatomic structure, the dental prosthesis model being generated accordingly.

After the insertion, in the anatomic context, of the computerized method based generated dental prosthesis, follow-up measurements are made at well-determined intervals of time, the behaviour of the prosthetic material being surveyed by computerized imagistic. The necessary statistics are generated in the same context of the expert type computerized environment MoDef, the statistical prediction being dually validated by the deformable model based behavioural prediction.

## 7. Conclusions and Future Work

Occlusal equilibration is a major step because besides motivating patients for a regular daily oral hygiene, it could significantly increase the longevity of FPR. More dental hygiene information should be given after prosthetic treatment and patients should be motivated to attend recalls on a regular basis for professional teeth-cleaning. Interdental cleaning aids should be explained and the patients have to be motivated to use them at least once a day and the using technique should be individualized.

We consider that patients should be motivated towards the importance of self-care and also of early self-diagnosis should be adjusted to the socioeconomic level and the education of the patients. Explaining to the patient the role he can play himself into the long-term success of a prosthetic treatment most often represents a particular financial effort and could motivate the patients towards a more important care and preoccupation towards orodental health and to a more careful daily individual oral hygiene, including the use of IDCAs.

Regarding the application of the deformable models theory, implemented in the context of the MoDef expert type software environment, it is known that the fact that modelling by advanced methods and techniques based on the deformable surfaces theory increases the efficiency of the dentofacial prosthetics procedures is a domain of great interest in the actual medical research.

## Figures and Tables

**Table 1:** Results reported in articles on FPR failure with similar including criteria [6].

Number	Cause	Number of abutment teeth in the study/number of affected abutment teeth	Mean incidence
1	Dental and root caries	3360/602 abutment teeth	18% of the abutment teeth
		1354/113 FPR	8% of the FPR
2	Endodontic treatment needed	2514/276 abutment teeth	11% of the abutment teeth
		1358/88 FPR	7% of the FPR
3	Decementing	1906/137 FPR	7% of the FPR
4	Aesthetic failure	1024/58 FPR	6%
5	Periodontal failure of the abutments	1440/62 FPR	4%
6	Fracture of abutments	1602/44 FPR	3%
7	Fracture of the bridge	1192/24 FPR	2%
8	Fracture of ceramic layer of metal-ceramic FPR	768/17 FPR	2%

**Table 2:** Periotest *C* mobility scores.

Clinical mobility	Periotest values
0	−08 up to +09
I	+10 up to +19
II	+20 up to +29
III	+30 up to +50

**Table 3:** The parameters found for evaluating the periodontal prognosis.

*a* (1) *S* = (sum of functional values of abutments) − (sum of functional values of missing teeth) (according to Table 1)	*a* (2) *L* = (length of the edentulous space)/(sum of the maximum *M*-*D* size of the missing teeth) ∗ 100	*a* (3) Plaque index	*a* (4) Gingival index	*a* (5) periodontal risk as assessed by Florida Probe software
≥0 *a*1 = −*S*∗5 pg = pg + 10; rii = rii + 1	10%–20% *a*2 = 100 − *L* pg = pg + 1	0–25% *a*3 = PI − 100 pg = pg + 4	0–25% *a*4 *=* GI pg = pg + 4	High *a*5 = 100 pg = pg − 4

<0 *a*1 = −*S*∗5 pg = pg − 10;	20–30% *a*2 = 100 − *L* pg = pg + 2	26–39% *a*3 *=* PI pg = pg + 3	26–39% *a*4 *=* GI pg = pg + 1	Medium *a*5 = 66 pg = pg − 3

	30–40% *a*2 = 100 − *L* pg = pg + 3	40–69% *a*3 *=* PI pg = pg − 2; rii = rii + 1; rcp = rcp + 1; rip = rip + 1	40–69% *a*4 *=* GI pg = pg − 2; rii = rii + 1; rcp = rcp + 1; rip = rip + 1	Low *a*5 = 33 pg = pg + 1

	>40% *a*2 = 100 − *L* pg = pg + 4	70–100% *a*3 *=* PI pg = pg − 4; rii = rii + 2; rcp = rcp + 2; rip = rip + 2	70–100% *a*4 *=* GI pg = pg − 4; rii = rii + 2; rcp = rcp + 2; rip = rip + 2	0 *a*5 = 0

**Table 4:** Resistance coefficients, according to the French school [Duchange, LeRiche].

	I.C.	I.L.	*C*.	Pm1.	Pm2.	*M*1.	*M*2.	*M*3.
Max.	2	1	3	4	4	6	6	2–5
Mand.	2	1	3	4	4	6	6	2–5

**Table 5:** The values of periodontal surfaces/according to Jepsen.

	I.C.	I.L.	*C*.	Pm1.	Pm2.	*M*1.	*M*2.	*M*3.
Max.	204	179	273	234	220	433	431	305
Mand.	154	168	268	180	207	431	426	373

**Table 6:** Coefficient found for mechanical resistance of FPR.

Name of the parameter	*a* (6) Static occlusion	*a* (7) Dynamic occlusion	*a* (8) Average degree of neighbouring teeth tilting	*a* (9) Esthetics of the studied area	*a* (10) Caries risk
Possible values	Functional *a*6 = 0 pg = pg + 2	Functional *a*7 = 0 pg = pg + 1	0°< *a* (8) ≤ 10° *a*8 = 0	Small gingival recessions ≤2 mm *a*9 = 0	High *a*10 = 100 pg = pg − 3; rii = rii + 2; rcp = rcp + 2; rip = rip + 2
	Minor bumps of the occlusal plane; *a*6 = 39 pg = pg + 1	1–3 interferences *a*7 = 40 pg = pg − 1	10°< *a* (8) ≤35° *a*8 = 66	2 < medium gingival recessions ≤6 mm *a*9 = 33	Average *a*10 = 66 pg = pg − 2; rii = rii + 1; rcp = rcp + 1; rip = rip + 1
	Medium bumps of the occlusal plane *a*6 = 69 pg = pg − 1;	1–3 premature contacts *a*7 = 40 pg = pg − 2	>35° *a*8 = 100	Important gingival recessions >6 mm *a*9 = 100	Low *a*10 = 33 pg = pg − 1
	Big bumps of the occlusal plane (±very deep or reversed Spee curve); *a*6 = 100 pg = pg − 2;	More than 3 interferences *a*7 = 100 pg = pg − 3			0 *a*10 = 0
		Peste 3 contacte premature; *a*7 = 100 pg = pg − 4			

**Table 7:** Failure causes [6].

Nr crt	Cause	Number of abutment teeth in the study/number of affected abutment teeth	Mean incidence (*z*)
*Z*1	Dental and root caries	3360/602 abutment teeth	18% of the abutment teeth
*Z*2		1354/113 FPR	8% of the FPR
*Z*3	Endodontic treatment needed	2514/276 abutment teeth	11% of the abutment teeth
*Z*4		1358/88 FPR	7% of the FPR
*Z*5	Decementing	1906/137 FPR	7% of the FPR
*Z*6	Aesthetic failure	1024/58 FPR	6%
*Z*7	Periodontal failure of the abutments	1440/62 FPR	4%
*Z*8	Fracture of abutments	1602/44 FPR	3%

**Table 8:** The weight of each peridodonal parameter.

Parameter	*a* (1) the sum of the functional values of the missing teeth − the sum of the functional values of the abutment teeth (according to Table 4)	*a* (2) (the length of the edentulous space)/(sum of the maximum *M*-*D* size of the missing teeth) ∗ 100	*a* (3) PI	*a* (4) GI	*a* (5) periodontal risk (assessed by Florida Probe software)
Weight of the index	*W*1 = *z*3 + *z*4 + *z*5 + *z*6 + *z*7 *K*1 = 7.15	*W*2 = *z*2 + *z*3 + *z*5 + *z*6 *K*2 = 8.33	*W*3 = *z*1 + *z*2 + *z*4 + *z*5 + *z*6 *K*3 = 11.11	*W*4 = *z*3 + *z*4 + *z*5 *K*4 = 6.75	*W*5 = *z*1 + *z*2 + *z*4 + *z*5 *K*5 = 9.92

**Table 9:** The weight of each mechanical parameter.

Parameter	*a* (6) Static occlusion	*a* (7) Dynamic occlusion	*a* (8) Average degree of neighbouring teeth tilting	*a* (9) Esthetics of the studied area	*a* (10) Caries risk
Weight index	*W*6 = *z*3 + *z*5 + *z*6 + *z*7 12.3	*W*7 = *z*3 + *z*5 + *z*6 + *z*7 12.3	*W*8 = *z*1 + *z*2 + *z*3 8.73	*W*9 = *z*1 + *z*2 + *z*4 + *z*5 + *z*6 11.11	*W*10 = *z*1 + *z*2 + *z*3 + *z*4 + *z*6 12.3

**Table 10:** The first FPR option generated by the algorithm.

*S*	3	6	0	FPR having as abutments 3 and 6 (13 and 16 according to FDI)
DL	4	5		Edentation of 4 and 5 (14 and 15 according to FDI)
*P*	0			The patient does not need removable denture or dental implants only

**Table 11:** Probability of failure in percentage due to each of the 9 causes (c1–c9) over time.

Time (years)	% of failure	c1	c2	c3	c4	c5	c6	c7	c8	c9
0.5	1.88	0.49%	0.26%	0.48%	0.04%	0.07%	0.25%	0.12%	0.16%	0.11%
1	3.45	0.91%	0.48%	0.88%	0.08%	0.13%	0.45%	0.23%	0.30%	0.20%
1.5	4.92	1.29%	0.68%	1.26%	0.11%	0.18%	0.65%	0.32%	0.43%	0.29%
2	6.33	1.66%	0.88%	1.62%	0.14%	0.23%	0.83%	0.42%	0.55%	0.37%
2.5	7.7	2.02%	1.07%	1.97%	0.17%	0.28%	1.01%	0.51%	0.67%	0.45%
3	9.03	2.37%	1.25%	2.31%	0.20%	0.33%	1.19%	0.59%	0.79%	0.53%
3.5	10.34	2.72%	1.43%	2.64%	0.23%	0.38%	1.36%	0.68%	0.91%	0.60%
4	11.63	3.06%	1.61%	2.97%	0.25%	0.42%	1.53%	0.76%	1.02%	0.68%
4.5	12.89	3.39%	1.79%	3.29%	0.28%	0.47%	1.69%	0.85%	1.13%	0.75%
5	14.14	3.72%	1.96%	3.61%	0.31%	0.52%	1.86%	0.93%	1.24%	0.83%
5.5	15.37	4.04%	2.13%	3.93%	0.34%	0.56%	2.02%	1.01%	1.35%	0.90%
6	16.59	4.36%	2.30%	4.24%	0.36%	0.61%	2.18%	1.09%	1.45%	0.97%
6.5	17.79	4.67%	2.47%	4.54%	0.39%	0.65%	2.34%	1.17%	1.56%	1.04%
7	18.99	4.99%	2.63%	4.85%	0.42%	0.69%	2.50%	1.25%	1.66%	1.11%
7.5	20.17	5.30%	2.80%	5.15%	0.44%	0.74%	2.65%	1.33%	1.77%	1.18%
8	21.35	5.61%	2.96%	5.45%	0.47%	0.78%	2.81%	1.40%	1.87%	1.25%
